# Mediator Effect of Cardiorespiratory Fitness on the Association between Physical Activity and Lung Function in Adults: Cross-Sectional Results from the Epimov Study

**DOI:** 10.3390/ijerph19159377

**Published:** 2022-07-31

**Authors:** Thatiane Lopes Valentim Di Paschoale Ostolin, Bárbara de Barros Gonze, Evandro Fornias Sperandio, Rodolfo Leite Arantes, Marcello Romiti, Victor Zuniga Dourado

**Affiliations:** 1 Department of Human Movement Sciences, Federal University of São Paulo (UNIFESP), Santos 11015-020, SP, Brazil; thatiane.ostolin@unifesp.br (T.L.V.D.P.O.); barbara.gonze@unifesp.br (B.d.B.G.); sperandio@unifesp.br (E.F.S.); 2 Department of Cardiovascular Medicine, Angiocorpore Institute of Cardiovascular Medicine, Santos 11075-350, SP, Brazil; arantes.r@uol.com.br (R.L.A.); marcello.romiti@angiocopore.com.br (M.R.); 3 Lown Scholars Program–Harvard T.H. Chan School of Public Health, Boston, MA 02115, USA

**Keywords:** physical fitness, spirometry, cardiopulmonary exercise test, vital capacity

## Abstract

We investigated whether cardiorespiratory fitness (CRF) mediates the association between moderate-to-vigorous physical activity (MVPA) and lung function in asymptomatic adults. We examined the cross-sectional results of 1362 adults aged 18–80 years from the Epidemiology and Human Movement Study. Participants were submitted to spirometry to obtain forced vital capacity (FVC) and forced expiratory volume in 1s (FEV1). Additionally, we used cardiopulmonary exercise testing to obtain peak oxygen uptake ($\dot{V}$O_2_) as a measure of CRF. Participants used a triaxial accelerometer for 4–7 days to obtain MVPA. Mediation analyses were performed considering the CRF as a mediator, MVPA as an independent variable, and FVC and FEV1 as dependent variables with adjustment for age, sex, and cardiovascular risk score. We aimed to investigate the total (path c) and direct (paths a, b, c’) effects through the regression coefficients. We also examined the indirect effect, which was obtained from the product of the coefficients (path ab). Our sample was composed mainly of overweight and middle-aged women. MVPA was positively related to CRF (path a), as well as CRF and lung function (path b). MVPA also presented a significant positive total effect (path c) in the lung function. However, this relationship became non-significant when CRF was included in the model for both FVC and FEV1 (path c’). We did not observe a direct effect of MVPA on the lung function. In contrast, the indirect effect was significant (path ab). Lastly, CRF mediated 60% of the total effect of MVPA on FVC and 61.9% on FEV1. CRF mediates the relationship between lung function and MVPA in asymptomatic adults. Therefore, our results reinforce the need to include CRF assessment in practice clinical routine and suggest that strategies focusing on CRF might be more promising to prevent respiratory diseases in adults.

## 1. Introduction

Since the prevalence of physical inactivity has increased worldwide [1,2], the literature has been recently focused on investigating the effect of meeting the minimum recommended moderate-to-vigorous physical activity (MVPA) and spending time in sedentary behavior on numerous health outcomes. The World Health Organization updated the physical activity (PA) guidelines by including recommendations for sedentary behavior and suggested performing more MVPA than recommended to avoid deleterious effects of high sedentary behavior [3]. However, cardiorespiratory fitness (CRF) is a physiological marker determined by genetic, functional, and behavioral factors [1,4,5,6]. Low CRF is associated with increased mortality risk, but high CRF is related to great survival, regardless of adjustment for the PA level [5]. Additionally, improving 1–2 metabolic equivalent of task (METs) in CRF reduces adverse effects of cardiovascular events and mortality risk [4,5]. Although heritage determined almost 50% of CRF [6], PA habits, especially MVPA, strongly influenced the other half [1,2,4,5]. Previously, the dose–response relationship between PA regimen and CRF was summarized according to type (e.g., running, cycling, climbing stairs), intensity (i.e., MVPA relative to subject’s capacity), frequency (i.e., 3–5 days per week of MVPA), time (i.e., session ≥ 10 min, 30–60 min per day, 150 min per week of MVPA), amount (i.e., recommendation of 500–1000 METs minutes per week), pattern (e.g., one continuous or multiple sessions per day), and progression (i.e., exercise volume gradually adjusted based on desirable goal) [5]. Therefore, it is necessary to establish goals for CRF combined with the recommendations of performing 150 min of MVPA per week [4]. The American Heart Association scientific statement [5] recommends that the clinical practice routine includes the CRF assessment, which allows a greater comprehension of the patients’ health and favors its management.

Only a few studies directly obtained MVPA and CRF, which introduce potential biases for the generalizability. Cardiopulmonary exercise testing (CPET) is the gold standard to obtain CRF (i.e., peak oxygen uptake obtained through CPET, $\dot{V}$O_2_) and provides other equally relevant clinical measures [5,7]. Although combining subjective and objective PA measures (i.e., questionnaires and accelerometers, respectively) may be more appropriate in research, triaxial accelerometry allows for a more precise and accurate measurement of MVPA [8,9,10]. Since triaxial accelerometry contributes to a better understanding of how much time was spent in PA at different intensities during the total wear-time, using the accelerometer reduces biases of recalling PA patterns and sedentary behavior and identifying PA intensity during daily living activities [9].

Chronic respiratory diseases are public health issues related to disability and mortality. Worldwide, the prevalence of chronic respiratory diseases has increased mainly due to smoking, exposure to environmental pollution, and physical inactivity [11,12,13]. PA is one of the pillars of chronic respiratory diseases treatment [11,14] since increasing PA is as effective as smoking cessation in preventing morbidity and mortality [11]. The Global Alliance against Chronic Respiratory Diseases encourages the implementation of health promotion (e.g., physical activity, diet and nutrition, traditional and complementary medicines) and chronic respiratory disease prevention policies [15]. According to Agusti and Farner [16], lung function abnormalities during child development or rapid declines in early adulthood could be related not only to behavioral and environmental factors (i.e., exposure to inhaling particles or gases) but also to numerous genetic factors [16]. However, the evidence is limited about the mechanisms of PA effects in adults with chronic respiratory diseases, suggesting possible mediation by physiological pathways [17]. Despite the fact that sports participation correlates to lung function, the type of activity is intimately associated with its effect [18]. Additionally, either insufficient MVPA or low CRF determine the decline in lung function [19,20,21,22]. Therefore, there are still gaps in chronic respiratory diseases etiology, especially regarding early changes in lung function in adults [11,16].

These relationships between PA and CRF and lung function have been investigated through multivariate methods, establishing significant predictors, and coping with confounders. Nevertheless, this approach presents some disadvantages, especially overfitting, conditioning on a shared effect (i.e., restricting attention to a certain level of some attribute that can be related to several causes), and the underestimating of causal effects (i.e., loss power, potential biases, attenuation, or suppression of real associations due to confounding) [23]. Despite the well-described relationship between lung function and both PA and CRF, the underlying mechanisms remain unclear. Therefore, it is reasonable to investigate if MVPA directly affects lung function or whether there is a pathway linking them (i.e., a possible CRF mediation role).

Mediation analysis emerges as an alternative for understanding these possible mechanisms and contributes to establishing the cause-and-effect, regardless of study design [24]. Accordingly, the analyses provide total, direct, and indirect effects between independent and dependent variables from each model if: (1) MVPA determines lung function, which attends the first requirement (i.e., the independent variable has to correlate significantly with the outcome); (2) CRF and MVPA are significantly associated, fulfilling the second requirement; and (3) CRF also influences lung function, ensuring that the mediator has to correlate with the outcome. After controlling the analysis by the mediator, the mediation can be considered complete or partial. Additionally, other confounders that affect lung function should be considered (for example, age, anthropometric measures, and sex [25]). To our knowledge, there is a lack of literature involving CRF, MVPA, and lung function from this perspective. Therefore, we hypothesized that CRF mediates the relationship between MVPA and lung function. In the present study, we aimed to investigate whether CRF is a mediator in the relationship between MVPA and lung function. Secondarily, we investigated the extent of this mediation.

## 2. Materials & Methods 

### 2.1. Study Design and Participants

We conducted a cross-sectional study from 2013 to 2016 with a convenience sample selected from the Epidemiology and Human Movement Study (EPIMOV Study). The EPIMOV Study is a prospective cohort study whose main purpose is to investigate the association between low PA and fitness levels and the development of chronic diseases, especially cardiovascular and musculoskeletal diseases [26].

Subjects from both sexes, aged 18 to 80 years and free from previously diagnosed pulmonary, cardiovascular, musculoskeletal, or neuromuscular diseases, were recruited through advertisements in social media, local universities, and newspapers. 

The EPIMOV Study excluded subjects with recent respiratory infections, abnormalities during CPET or spirometry, and refusal to participate. In the present study, we included only participants with valid triaxial accelerometry data. Participants with difficulty in comprehending or performing the study protocol or those with alterations or operational problems during the CPET (i.e., potentially lethal arrhythmias, signals of suggestive myocardial ischemia, chest pain, and submaximal effort) were excluded. 

All participants provided written informed consent before participation. The Ethics Committee of the Federal University of São Paulo approved the present study (#186.796).

### 2.2. Study Measurements of the EPIMOV Study

The EPIMOV Study protocol was performed during two visits a week apart. On the first visit, we carried out a clinical health screening followed by an anthropometric evaluation, and CPET (Quark PFT, COSMED, Pavona, Italy). Lastly, we provided instructions for triaxial accelerometer-based habitual PA level (Actigraph GT3x +, MTI, Pensacola, FL, USA) during 7 consecutive days starting from first visit. On the second visit, participants returned the devices. Then, they underwent body composition assessment (310e, Biodynamics, Baltimore, USA), among other measurements from EPIMOV Study protocol, including isokinetic muscle function and a six-minute walk test. The first and second visits were located at Angiocorpore Cardiovascular Medicine Institute and EPIMOV Laboratory, respectively. In the present study, we analyzed the results of the following assessments.

### 2.3. Clinical Health Screening 

Participants underwent a clinical health screening, including age, sex, educational level, long-term use of medication, and current diagnosis of diseases. Then, we obtained risk factors for cardiovascular disease by self-report as follows: age (male ≥ 45 years; female ≥ 55 years), family history of cardiovascular diseases (myocardial infarction or sudden death in first-degree relatives), arterial hypertension, diabetes, dyslipidemia, obesity, and current smoking. Lastly, we calculated the 10-year cardiovascular risk score (CVRS) based on sex, age, BMI, systolic blood pressure (treated and untreated), smoking, and diabetes as recommended [27,28].

### 2.4. Anthropometric Evaluation and Bioelectrical Impedance

We obtained height (m) and weight (kg) using a digital scale with a stadiometer. Then, we calculated body mass index (BMI). Participants were classified as obese when BMI > 30 kg/m^2^.

We determined body composition through the bioelectrical impedance (310E BIODYNAMICS, Detroit, MI, USA) according to previously described [29]. We calculated lean and fat body masses based on the regression equation developed for healthy subjects [30]. The data were registered for further analysis and expressed in absolute value and percentage.

### 2.5. Spirometry

Spirometry was performed using a calibrated spirometer (Quark PFT, Cosmed, Pavona di Albano, Italy) according to the criteria established by the American Thoracic Society [31]. We obtained forced vital capacity (FVC), forced expiratory volume in 1s (FEV1) and then determined the FEV1/FVC ratio. We calculated the predicted values using national equations. Participants with obstructive ventilatory disorder were excluded from the present study [32]. 

### 2.6. Cardiopulmonary Exercise Testing

Participants were submitted to a CPET on a treadmill under a ramp protocol (ATL, Inbrasport, Porto Alegre, Brazil). Initially, the participant remained at rest period for 3 min followed by an individualized incremental protocol. Briefly, speed and grade started at 3 km/h and 0%, respectively, and were automatically increased in a linear and individualized way based on $\dot{V}$O_2_ max predicted by using the Vivacqua and Hespanha equation [33]. The prediction included the body mass, age, sex, and level of PA of each individual. The Inbramed software calculated, designed, and applied the equation for the ramp protocol, i.e., to make the participant reach $\dot{V}$O_2_ max, on average, in 10 min. We obtained ventilatory, cardiovascular and metabolic variables breath-by-breath using a gas analyzer (Quark PFT, Cosmed, Pavona, Italy) and a 12-lead ECG (C12x, Cosmed, Pavona, Italy) during the test. The data were filtered every 15 s for further analysis. The peak $\dot{V}$O_2_ was considered the average value obtained in the last 15 s at the peak of the incremental exercise, being expressed as an absolute value (mL/min), corrected by weight (mL/min/Kg) and as percentage of predicted (% pred.) [34,35]. 

We discontinued the test in cases of potentially lethal arrhythmias, hyperreactive systolic blood pressure response (i.e., >250 mmHg), signs or symptoms suggestive of myocardial ischemia, or the participant’s request. The tests were considered as maximal effort when they filled at least two of the following parameters: heart rate ≤ 10 bpm under the maximal heart rate predicted or 5% under than the value obtained with the equation (220-age), respiratory exchange ratio (carbon dioxide output/$\dot{V}$O_2_) > 1.0 [36,37].

### 2.7. Triaxial Accelerometry

We evaluated the PA level through triaxial accelerometers (Actigraph GT3x +, MTI, Pensacola, FL, USA) previously validated [38,39,40]. Participants wore the device at the waist above the dominant hip for at least ten waking hours a day in seven consecutive days of assessment during their usual waking hours, i.e., until bedtime, except in the shower and water-related activities. The device can measure the quantity of PA while the subject is performing household activities (e.g., laundry, dishwashing, moving a small load, and vacuuming) and locomotive activities (e.g., slow, normal, or brisk walking, walking while carrying a bag, jogging, or running, cycling, among others) according to thresholds based on counts per minute (cpm) and MET as described below. We considered as a valid measure for inclusion, in the final analysis, the data of those participants who used the device for at least four days in a minimum of three weekdays and one weekend day. We considered non-wearing time as an interval of zero counts for 60 or more minutes. Both non-wearing time and the thresholds for the intensity of the PA were evaluated as previously described.

The thresholds for the sedentary behavior and the intensity of PA were as follows: sedentary behavior (<100 cpm, ≤1.5 MET); light-intensity (<1951 cpm, <3 METs); moderate-to-vigorous (>1951 cpm, ≥3 METs). We considered those participants with less than 150 min per week of MVPA or less than 75 min per week of vigorous PA as physically inactive [8]. Since the PA recommendations are based on minutes per week, we registered sedentary behavior, light-intensity PA, moderate PA, vigorous PA, and MVPA as minutes per week for further analysis and % of total wear-time.

### 2.8. Statistical Analysis

Statistical analysis was performed by using STATA, version 14. The data were analyzed descriptively and expressed as mean and standard deviation for continuous variables and as frequency and percentage for categorical variables. 

**Figure 1:**
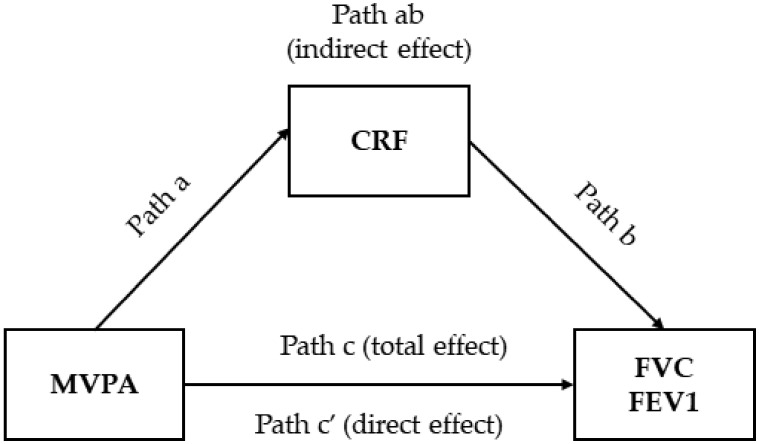
Cardiorespiratory fitness structural equation models of the relationships between moderate-to-vigorous physical activity and lung function. MVPA, moderate-to-vigorous physical activity; CRF, cardiorespiratory fitness; FVC, forced vital capacity; FEV1, forced expiratory volume in 1 s.

We conducted a mediation analysis to investigate whether the relationship between MVPA and lung function (e.g., FVC and FEV1) was mediated by CRF. In the present study, the peak $\dot{V}$O_2_ (mL/min/kg) was used as a mediator, MVPA (% of total) as independent variable and FVC (% pred.) and FEV1 (% pred.) as dependent variables. We included age, sex, and Framingham CVRS as covariates. We estimated the total, direct and indirect effects of MVPA on lung function (Figure 1). 

We used structural equations-modeling frameworks to examine the mediating role of CRF in the relationship between MVPA and lung function as shown in Figure 1. The aim was to investigate the total (*path c*) and direct (*path c’*) effects through the regression coefficients and significance between each model’s independent and dependent variables. We also examined the indirect effect, which was obtained from the product of the coefficients (*path ab*) (Figure 1). 

The total effect (*path c*) represents the correlation between PA and lung function without considering the mediating potential. After the structural equation modeling elaboration, which considered the CRF as a potential mediator, we can observe the effect *a*, which represents the correlation between the independent variable and the mediator (i.e., MVPA and CRF). The effect *b* represents the correlation between the mediator and the dependent variable (i.e., CRF and lung function). After adjustment by confounders [41], we can conclude that the *path ab* represents the indirect effect of PA on lung function, i.e., the effect mediated by the CRF in this example. The mediated effect is, simply, the multiplication of effects found in *paths a* and *b*. Therefore, the direct effect *c’* represents the effect of the independent variable after considering the mediated effect, i.e., *ab-c* [42] (Figure 1).

Conducting a mediation analysis in the present study requires: (1) the independent causal variable (MVPA) has to correlate significantly with the dependent variable (FVC and VEF1) (significant total effect, *path c*); (2) the independent variable (MVPA) has to correlate significantly with the mediator (CRF) (*path a*); (3) the mediator (CRF) has to correlate significantly with the dependent variable (FVC and VEF1); and (4) the mediation is complete when the direct effect *c’* is zero and became non-significant when controlled by the mediator.

The mediation analysis was performed based on all available data (*n* = 1362) and using full-information maximum likelihood, which allows valid inferences assuming that the data are missing at random.

In order to increase the statistical power of the multivariate models, we calculated the Framingham CVRS. We did a sensitivity analysis and, due to the strength of the correlation, we chose the CVRS as a covariate instead of inserting each cardiovascular risk factor. Briefly, CVRS expressed as a percentage can be calculated based on sex, age, SBP, treatment for high blood pressure, current smoking, diabetes, and BMI. Also, we checked the correlations between chosen covariates to avoid multicollinearity. Since we found no strong correlations among then, we were able to include age, sex and CVRS in the same model, despite the use of age and sex in the CVRS. Considering the fundamental influence of height on lung function variables, this was also considered for adjustment in the models in which FEV1 and FVC were the dependent variables. We evaluate the fit of the models by calculating the coefficients of determination of the equations (R^2^), as well as calculating the values of the Comparative Fit Index, for which we consider values above 0.9 as evidence of adequate adjustment.

We evaluated model fit with the comparative fit index, where we considered values above 0.9 as evidence of adequate fit. Lastly, we used the Sobel–Goodman test to investigate the proportion of total mediated effects. Complete mediation was considered in the case where *path c’* was non-significant (approximately zero). As a general rule, a complete mediation is considered only if *b/c* > 0.80 and partial mediation in cases where the influence of the independent variable on the dependent variable (*path c’*) has been reduced, but still remains significant and different from zero to introduce the mediator [42]. We set the probability of alpha error in 5%.

## 3. Results

**Figure 2:**
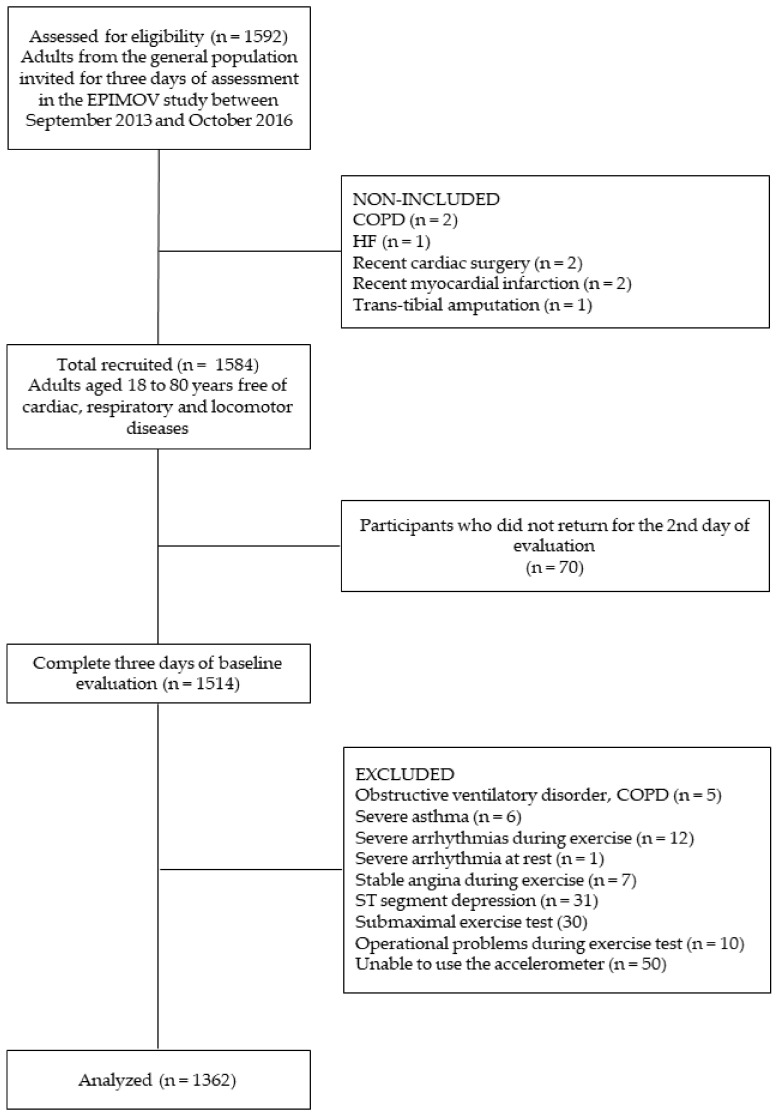
Flowchart of the study. EPIMOV: The Epidemiology and Human Movement Study. COPD: chronic obstructive pulmonary disease. HF: heart failure.

We considered 1592 participants eligible from the EPIMOV Study, but 1362 (541 males and 821 females) were included in the present study (Figure 2).

Our sample was composed mainly of overweight and middle-aged women (46.7 ± 14.2 years). We observed a low prevalence of self-reported hypertension, but a high prevalence of physical inactivity and obesity. The prevalence of diabetes and current smoking was similar to values observed in the Brazilian population (Table 1).

Thirty-four percent of participants reported regular use of medication. The most commonly reported medications were classified as biguanides, betablockers, angiotensin receptor antagonists, statins, angiotensin converting enzyme inhibitors, and thiazide diuretics.

As can be seen in Table 1, our participants reached, on average, 102.2% peak $\dot{V}$O_2_ pred., suggesting exercise tolerance and regular-to-good CRF. Regarding CPET data, we included in our analysis only valid and maximal tests. According to sex, males presented peak $\dot{V}$O_2_, on average, 40.1 ± 11.0 and females, 26.7 ± 8.5. Maximal heart rate was, on average, 171 ± 16 (94.6 ± 6.9% pred.) and 162 ± 19 (92.3 ± 7.9% pred.), respectively, for males and females. Lastly, the respiratory exchange ratio was higher than 1.0 for both males and females (1.18 ± 0.10 and 1.11 ± 0.12, respectively). 

Among our participants, we obtained 1040 (76.3%) valid accelerometry data. Almost 30% of our sample was classified as physically inactive (*n* = 368, 27%). The PA level in detail can be seen in Table 2.

**Figure 3:**
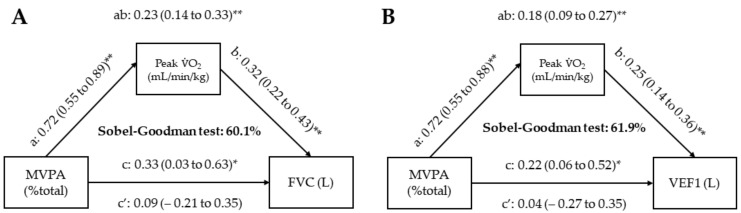
Cardiorespiratory fitness structural equation models of the relationships between moderate-to-vigorous physical activity and lung function, adjusted for age, sex, height, and cardiovascular risk score. (**A**) Results from mediation analysis with forced vital capacity as dependent variable. (**B**) Results from mediation analysis with forced expiratory volume in 1s as dependent variable. * *p* < 0.05; ** *p* < 0.01. FVC, forced vital capacity; FEV1, forced expiratory volume in 1 s; MVPA, moderate-to-vigorous physical activity.

The mediation analysis was presented in Figure 3. As expected, MVPA was positively related to CRF (*path a*) in both A and B. In turn, CRF was associated with both FVC (A) and VEF1 (B) (*path b*). MVPA also presented a significant positive total effect (*path c*) in FVC (A) and VEF1 (B). However, these relationships became non-significant for FVC (A) and FEV1 (B) (*path c’*) when controlled by CRF. Therefore, we did not observe a direct effect of MVPA on the lung function (*path c’*). In contrast, the indirect effect was also significant (*path ab*) in A and B. Lastly, the Sobel–Goodman test confirmed that CRF mediated 60% of the total effect of MVPA on FVC and 61.9% on FEV1 (Figure 3).

## 4. Discussion

We investigated the role of CRF as a mediator in the relationship between MVPA and lung function. Our findings showed that the CRF largely mediates the relationship between MVPA and lung function in asymptomatic adults. To our knowledge, this is the first study to apply mediation analysis to understand the pathways linking lung function, PA level, and CRF. Therefore, health promotion should emphasize goals for lifestyle changes that help to maintain or improve CRF. In addition, our findings corroborate the need for assessing and monitoring CRF in clinical settings.

Our participants were mostly overweight and middle-aged women with low prevalence of arterial hypertension, but a high prevalence of physical inactivity and obesity. These findings agree to recent studies that showed increased rates of inactivity and obesity over the past few years [1,5,43]. We found similar prevalence of diabetes and smoking reported for the Brazilian population (Table 1).

As expected, MVPA was positively related to CRF. A previous cross-sectional study showed a moderate correlation between MVPA and CRF [44]. Chronic obstructive pulmonary disease (COPD) patients are mostly insufficiently active and spend more than 60% of the day sitting or lying [45]. CRF, six-minute walking distance, and maximal workload presented a moderate correlation with walking time and standing time in patients with COPD [45]. Similar results were found when compared to European patients with COPD, except the Brazilian patients were more active [46,47]. Moreover, CRF can be improved as a result of changes in MVPA and sedentary behavior [44].

Our results showed that CRF was positively related to CVF and FEV1. Further from its association with mortality risk, CRF presents a strong association with lung function, but also with respiratory and peripheral muscle strength [48]. The results from a previous study showed that each additional minute of treadmill duration was related to 1.00 mL/year and 1.55 mL/year less decline, respectively, in FEV1 and FVC. In addition, when higher decreased CRF, higher lung function declines [20].

We found a positive total effect of MVPA in CVF and FEV1. A cohort study with 1329 Norwegian adults with asthma found 1.5–2.1% less decline in FEV1 and FEV1/FVC ratio in active subjects compared to those inactive in an 11-year follow-up [49]. The Copenhagen City Heart Study showed that moderate and vigorous PA attenuated the lung function decline and reduced the COPD incidence in active smokers in 11-year follow-up [19]. However, PA was not related to lung function decline in young adults or subjects with mild COPD [19]. Additionally, the PA’s effect was higher in subjects with asthma [19]. Previously study showed that sports affect lung function, but the extent of this association was linked to type of activity, especially endurance sports [18].

However, we did not observe a direct effect of MVPA on the lung function since the relationship became non-significant when CRF was included. CRF mediated 60% of the total effect of MVPA on FVC and 61.9% on FEV1 (Figure 3). Our results are similar to previous studies [44]. CRF explained 73% of the variance in the relationship between MVPA and cardiometabolic risk [44] in a cross-sectional design. Given the mediating role of CRF, a previous study [44] already suggested that CRF should be one of the targets of lifestyle interventions. Knaeps et al. [48] evaluated 652 subjects with 10-year follow-up and observed decreased CRF, increased sedentary behavior, and maintenance of MVPA. The 10-year change CRF mediated the relationship between sedentary behavior, MVPA, and cardiometabolic risk [48]. The decreased CRF was associated with a higher increase in all cardiometabolic risk markers, changes in cardiometabolic risk, and other individual markers and were twice as strong as changes in sedentary behavior and MVPA [48].

Although physically active subjects with low CRF and inactive with high CRF present a similar risk of all-cause mortality, Lu et al. [50] suggested that high CRF does not guarantee a lower mortality risk in physically inactive elderly. Notwithstanding, Davidson et al. [51] investigated the independent and associated effects of CRF and physical activity on mortality risk in 1349 men aged 20–89 years with 8-year follow-up. When separately considered, the increase of each 1 MET in CRF was related to a decrease of 15% in mortality risk, while being active (e.g., ≥150 min of MVPA per week) presented a decrease of 17% [51] However, PA did not remain a significant predictor of mortality after adjustment for CRF [51]. These previous results also corroborate our findings since the PA may play an indirect effect that should be confirmed.

The mediating role of CRF is probably due to its genetic determination and responsiveness to lifestyle change and exercise training. Almost 80% of the patients undergoing cardiac rehabilitation phase II improved CRF an average of 2 METs in 10-year follow-up, regardless of age, sex, and baseline fitness status [52]. Additionally, these authors showed that both patients with low and regular baseline CRF improved after cardiac rehabilitation [52]. Accordingly, the lung function declines proportionally to CRF with advancing age, but it is less responsive to endurance training than $\dot{V}$O_2_ [53]. Therefore, our results agree with recent literature, corroborating the need to address CRF assessment and goals to its maintenance or improvement as an essential part of health screening and rehabilitation.

The main weakness of our study was the cross-sectional design. Although the self-reported cardiovascular risk was a weakness, the study had a sufficient sample for the proposed statistical analysis. Moreover, several epidemiological studies commonly use self-reported measures. One of our strengths was the similarity between our data and the Brazilian population’s cardiovascular risk prevalence, except for physical inactivity. However, this may be explained by methodological differences since we obtained PA level through the triaxial accelerometry [54]. Despite its advantages, we need to address some limitations of triaxial accelerometer; for instance, inability to detect stationary movement or determine the activity context (leisure time and work) and misclassification related to water activities, contact sports, cycling, or seasonable variations [10,54]. Although using a triaxial accelerometer do not allow for reliable data on aquatic activities or cycling, the region in which the study was performed favors active transportation and has fewer weather condition impacts in PA level compared to places with extreme weather conditions.

Despite the study design not allowing for the establishment of cause-and-effect, mediation analysis provides an assessment of the mechanisms and a certain causality since it analyzes potential pathways linking exposure and effect. In addition, mediation analysis has become common in epidemiology in order to identify the direct and indirect effects of exposure in relation to an outcome [55]. The use of mediation analysis is feasible and adequate to investigate the relationship between MVPA and lung function mediated by CRF. Furthermore, to our knowledge, this is the first study to investigate the role of CRF as the mediator of the relationship between MVPA and lung function, especially because we consider CRF to be a mediator rather than confounding as previous studies. Moreover, we obtained direct measurements of CRF and MVPA respectively through the CPET and triaxial accelerometry. A previous study suggests that the CPET is more sensitive than spirometry to early diagnosis of incipient ventilatory changes [56], which further strengthens the measures performed in our study and reinforces the applicability of the CRF assessment in clinical practice.

Our study has practical implications for patients, health professionals, stakeholders, policy makers and researchers. First, our findings confirm a pathway linking CRF, MVPA and lung function. In clinical settings, our study corroborates the recommendations from American Heart Association about the inclusion of CRF assessment in clinical practice routine either through CPET or validated field walking tests or even using algorithms and non-exercising variables. Besides showing the importance of assessing and monitoring CRF, we reinforce the need of establishing goals not only for lifestyle changes, but also pointed out prioritizing activities that favors maintenance or improvement of CRF. In future studies, we are interested in analyzing prospectively the role of CRF as a mediator in the relationship between MVPA and lung function. Nevertheless, whether CRF can play the role of mediator in the relationship between other PA intensities (e.g., light-intensity PA) and lung function or even between sedentary behavior and lung function remains unknown and could be further addressed.

## 5. Conclusions

CRF mediates more than 60% of the relationship between MVPA and lung function in asymptomatic middle-aged adults. Preventive strategies that prioritize CRF may be more promising to prevent respiratory diseases and reinforces the importance of adding the CRF assessment in clinical practice routine. Future studies should address this mediation prospectively.

## Figures and Tables

**Table 1:** General characteristics of the studied sample (*n* = 1362).

Variables	
Age (years)	46.7 ± 14.2
Sex, n (%)	
Male	541 (39.7)
Female	821 (60.3)
Anthropometry and body composition	
Weight (kg)	76.9 ± 17.2
Height (m)	1.64 ± 0.09
Body Mass Index (kg/m^2^)	28.4 ± 6.0
Lean Body Mass (% of total)	69.0 ± 9.1
Fat Body Mass (% of total)	30.8 ± 9.1
Cardiovascular risk factors, n (%)	
Hypertension ¥	271 (19.9)
Diabetes ¥	127 (9.3)
Dyslipidemia ¥	1293 (30.9)
Obesity €	496 (36.4)
Current smoking ¥	150 (11.0)
Physical inactivity £	368 (27.0)
Framingham CVRS	44.0 ± 18.0
Cardiopulmonary exercise testing	
Peak oxygen uptake (mL/min)	2385 ± 881
Peak oxygen uptake (mL/min/Kg)	32.1 ± 11.6
Peak oxygen uptake (% pred.)	102.2 ± 20.4
Spirometry	
FVC	95.3 ± 13.3
FEV1 (% predicted)	94.8 ± 14.0
FEV1/FVC (%)	81.3 ± 6.0
RVD defined by spirometry, n (%)	136 (10)

Data were expressed as mean ± SD or frequency (%). CVRS: cardiovascular risk score; FEV1: forced expiratory volume in 1 s; FVC: forced vital capacity; RVD: restrictive ventilatory disease. ¥ Assessed through self-report. € Classified according to Body Mass Index (>30 kg/m^2^) after anthropometric evaluation. £ Assessed through triaxial accelerometry.

**Table 2:** Accelerometer-based physical activity level (*n* = 1362).

Accelerometer-Based Activity	Minutes/Week	% Total
Sedentary behavior	3779.55 ± 1403.11	72.2 ± 8.5
Light-intensity physical activity	1161.80 ± 527.80	22.5 ± 7.5
Moderate physical activity	242.77 ± 153.52	4.7 ± 2.6
Vigorous physical activity	20.04 ± 46.73	0.38 ± 0.93
Very vigorous physical activity	2.91 ± 11.60	0.06 ± 0.22
Total MVPA	265.73 ± 171.45	5.1 ± 2.9

## Data Availability

The datasets used and/or analyzed during the current study are available from the corresponding author on reasonable request.

## Abbreviation

COPD	chronic obstructive pulmonary disease
CRF	cardiorespiratory fitness
CVRS	cardiovascular risk score
EPIMOV	Epidemiology and Human Movement Study
FEV1	forced expiratory volume in 1 s
FVC	forced vital capacity
MET	metabolic equivalent of task
MVPA	moderate-to-vigorous physical activity
PA	physical activity
$\dot{V}$O_2_	oxygen uptake
